# P032. Coenzyme Q-10 and migraine: a lovable relationship. The experience of a tertiary headache center

**DOI:** 10.1186/1129-2377-16-S1-A139

**Published:** 2015-09-28

**Authors:** Ennio Pucci, Luca Diamanti, Silvano Cristina, Fabio Antonaci, Alfredo Costa

**Affiliations:** Department of Brain and Behavioral Sciences, University of Pavia, Pavia, Italy; IRCCS National Neurological Institute, “C. Mondino”, Pavia, Italy; Headache Science Center, University Consortium for the Study of Adaptive Disorders and Headache (UCADH), Pavia, Italy

## Background

Coenzyme Q-10 (ubiquinone) is a small hydrophobic substance that acts as an electron carrier in the mitochondrial respiratory chain. Its main activity is to protect DNA, proteins and lipids from oxidative stress. In the literature, a role of brain oxidative metabolism in the pathogenesis of migraine has been hypothesized [1]. Few clinical trials are described using coenzyme Q-10 in migraine prophylaxis, even in pediatrics [2–4]. The aim of this work was to present our experience of migraine prevention, prescribing coenzyme Q-10 to 20 adult patients with migraine without aura.

## Materials and methods

Patients were enrolled in a tertiary headache center and followed for a period of 60 days (visit 1 and visit 2). The dose of coenzyme Q-10 was 200 mg/day. Visual analogue scale (VAS) was used to measure pain.

## Results

In our cohort, male/female ratio was 1:5, while the mean age was 32.1 years (range, 22-49 years). Patients had a relatively short history of disease (mean 5.6 years; range 2-18), indeed only 2 of them were on a first-line treatment whereas coenzyme Q-10 was the starting therapy for others. We noticed a significant reduction of the number of crises at visit 2 (mean 3.15 vs 0.9, p < 0.05), as well as VAS score (mean 6.65 vs 1.45, p < 0.05) and monthly days of headache (mean 6.3 vs 1.5, p < 0.05). No one showed side effects, body weight did not vary (mean 56.55 vs mean 56.65) and patients did not even experience drastic weight loss or gain. The drug was well tolerated with a mean satisfaction score of 7.65 (range, 0-10). Moreover, patients reported positive effects on fatigue.

## Conclusions

Coenzyme Q-10 is a safe and effective therapy for migraine prophylaxis.

Written informed consent to publish was obtained from the patient(s).

## Conflict of interest

None. This study did not receive any industry funding.
